# Long-read sequencing of the human cytomegalovirus transcriptome with the Pacific Biosciences RSII platform

**DOI:** 10.1038/sdata.2017.194

**Published:** 2017-12-19

**Authors:** Zsolt Balázs, Dóra Tombácz, Attila Szűcs, Michael Snyder, Zsolt Boldogkői

**Affiliations:** 1 Department of Medical Biology, Faculty of Medicine, University of Szeged, Szeged 6720, Hungary; 2 Department of Genetics, School of Medicine, Stanford University, Stanford, California 94305, USA

**Keywords:** Herpes virus, Transcriptomics, RNA sequencing

## Abstract

Long-read RNA sequencing allows for the precise characterization of full-length transcripts, which makes it an indispensable tool in transcriptomics. The human cytomegalovirus (HCMV) genome has been first sequenced in 1989 and although short-read sequencing studies have uncovered much of the complexity of its transcriptome, only few of its transcripts have been fully annotated. We hereby present a long-read RNA sequencing dataset of HCMV infected human lung fibroblast cells sequenced by the Pacific Biosciences RSII platform. Seven SMRT cells were sequenced using oligo(dT) primers to reverse transcribe poly(A)-selected RNA molecules and one library was prepared using random primers for the reverse transcription of the rRNA-depleted sample. Our dataset contains 122,636 human and 33,086 viral (HMCV strain Towne) reads. The described data include raw and processed sequencing files, and combined with other datasets, they can be used to validate transcriptome analysis tools, to compare library preparation methods, to test base calling algorithms or to identify genetic variants.

## Background & Summary

Long-read sequencing surveys of eukaryotic transcriptomes have demonstrated the potential of this new technology in identifying novel transcripts and characterizing transcript isoforms^1–3^. The currently available long-read sequencing platforms have a relatively high error-rate and a low throughput. However, even in its present state, long-read RNA sequencing (RNA-Seq) is well suited for the characterization of smaller transcriptomes of organisms with known reference genomes, such as viruses^4–6^. However, only few long-read RNA-Seq datasets are currently available, therefore our understanding of the characteristics and limitations of this technology is still lacking. More transcriptomic data generated by long-read sequencing would also facilitate the development of analysis tools needed to evaluate such data.

The Human cytomegalovirus (HCMV) is a human pathogenic betaherpesvirus with a genome size of approximately 235,000 base pairs (bp). Northern blot and, more recently, Rapid Amplification of cDNA Ends (RACE) analyses have been utilized to characterize HCMV transcripts^7^. A recent Illumina-based short-read sequencing study has shown that the HCMV transcriptome is more complex than it had been recognized previously^8^. However, due to technical limitations, much of the HCMV genome remained transcriptionally unannotated^7^.

**Figure 1:**
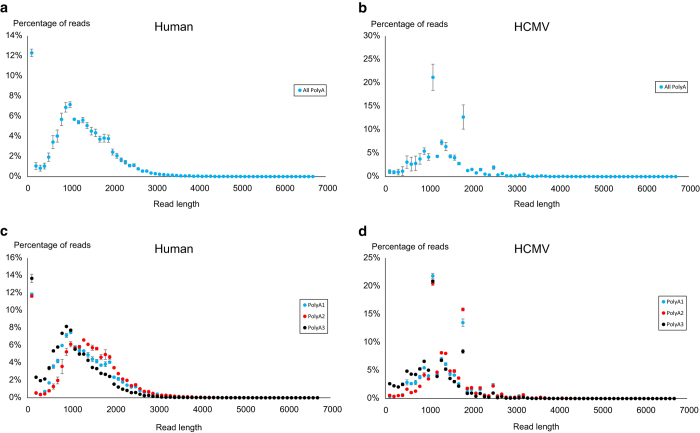
The distribution of read lengths in the Poly(A)-selected samples. The average distribution of read lengths which align to the human (hg19) genome is shown in **a** (*n*=7), and for the HCMV genome (FJ616285.1) in **b** (*n*=7). The same can be seen broken down to the three sample complexes in **c** and **d** (for the hg19 and the FJ616285.1 genomes respectively). The sample complex PolyA1 was used for three SMRTcells, PolyA2 and PolyA3 were used for two SMRTcells each. Error bars represent s.e.

We sequenced eight cDNA libraries, prepared from HCMV-infected fibroblast cells, with a Pacific Biosciences RSII sequencer to characterize the lytic HCMV transcriptome. To be able to capture transcripts with different expression kinetics, we pooled isolated total RNA from eight different post infection time points (1, 3, 6, 12, 24, 72, 96 and 120 h). Seven sequencing runs were carried out using oligo(dT) selection methods, to analyse the polyadenylated fraction of transcripts and one library was prepared by random primer amplification to capture non-polyadenylated transcripts as well. Our aim with these experiments was to assess the utility of Pacific Biosciences isoform sequencing (Iso-Seq) sequencing in the transcriptome profiling of HCMV, to identify novel viral transcripts and to complement the already existing viral transcriptome^9^. Here, we provide an overview of the library preparation methods used and a detailed description of the raw (Table 1) and the pre-processed data (Tables 2–4). The data contain 156,390 reads, 33,086 of which map to the HCMV (FJ616285.1) genome. As the pooled samples also contained RNA from early post infection time points, when host transcription has not yet been disrupted by the virus, most of the reads (122,636 reads) aligned to the human genome. The average read lengths aligning to the human and the HCMV genomes are 1,048 and 1,168 bp respectively, however the reads in the random-primer-amplified samples are generally shorter. Altogether 28,661 high-quality (>0.99) isoforms could be determined using the IsoSeq cluster routine. The seven poly(A)-selected sequencing runs are all technical repetitions, prepared from the same cDNA library, however, before loading onto the SMRTcells, three separate sample complexes were prepared. Table 2 shows that the sequencing yields can be rather different from the same library, but shows much less variation from the same sample complex. The read length distribution of the samples is visualized in Fig. 1.

## Methods

These methods are expanded versions of descriptions in our related work^9^.

### Cells cultures and viral infection

Eight T75 cell culture flasks (Thermo Fischer) of human embryonic lung fibroblast cells (MRC-5, ATCC CCL-171) were grown at 37 °C and 5% CO_2_ in low-glucose DMEM supplemented with 10% FBS (Gibco Invitrogen), and 100 units of potassium penicillin and 100 μg of streptomycin sulphate per 1 ml. The medium was removed from the rapidly-growing semi-confluent MRC-5 cells and 2 ml of no-glucose DMEM containing HCMV Towne strain was added. The virus stock was obtained from the American Type Culture Collection (ATCC). The cells were incubated with the virus-containing solution for 1 h at a multiplicity of infection of 0.05 plaque-forming units per cell. The virus suspension was then removed and washed with PBS. Subsequently, the cells were incubated in fresh culture medium for 1, 3, 6, 12, 24, 72, 96 or 120 h.

### RNA extraction and cDNA library preparation

The NucleoSpin^®^ RNA kit (Macherey-Nagel) was used to isolate RNA from all eight flasks (one for each time point). 10–10 μl isolated total RNA solution of each sample was taken and pooled before using the Oligotex mRNA Mini Kit (Qiagen) to select polyadenylated RNA, 23 ng of which was reverse transcribed with anchored oligo(dT) primers. 1–1 μl isolated total RNA solution of each sample was pooled and the rRNA was depleted by RiboMinus Eukaryote System v2 (Ambion) kit. The residual 2 ng RNA was reverse transcribed by random primers. No size selection has been performed on any of the samples. To maximize the performance of the SMRTcell, Run3 contained random selected cDNA samples from pseudorabies virus (PRV) infected PK-15 cells pooled together with the HCMV sample. The growth conditions and RNA extraction methods for this experiment followed the same protocols as described in our earlier article^5^. Runs 7 and 8 contained gDNA libraries of PRV, grown on PK-15 cell line. These libraries were prepared as described previously^10^.

### SMRTbell template preparation and SMRT sequencing

cDNA production and SMRTbell library preparation were carried out according to the PacBio Iso-Seq protocol, using the Clontech SMARTer PCR cDNA Synthesis Kit. The cDNA was amplified through 18 cycles. SMRTbell template libraries were prepared using 500 ng of amplified cDNA sample with the PacBio DNA Template Prep Kit 2.0. Annealing of the sequencing primer and binding polymerase P6 to the SMRTbell templates were performed according to the recommendations of the PacBio calculator. The polymerase-template complexes were bound to MagBeads, loaded onto SMRTcells and sequenced on the PacBio RS II sequencer. Briefly, the sequencing primer was diluted to 150 nM in PacBio Elution Buffer (EB). The annealing reaction was carried out with 1 μl library DNA (cc: 24 ng μl^−1^), the diluted primer and 10x primer buffer. The final concentration of the mixture was 0.8333 nM. Annealing was performed at 80 °C for 2 min then the temperature was ramp to 25 °C at a rate of 0.1 °C per sec. DNA polymerase was diluted to a final concentration of 50 nM in Binding Buffer v3 (BB). Diluted polymerase was bound to the annealed template with the following components: dNTP, DTT and BB. The final concentration of the complex was 0.5 nM and it was incubated at 30 °C for 4 h. 0.5 μl from the sample complex and 18.5 μl MagBead Binding Buffer were mixed (the final concentration was 0.0125 nM). MagBeads were prepared in short, as follows: 73.9 μl MagBeads were washed with 73.9 μl MagBead Wash Buffer, then 73.9 μl MagBead Binding Buffer was added. The sample complex was bound to the washed, prepared MagBeads for loading to the RSII sequencer: sample complex (19 μl) was added to the beads, and then it was incubated in a rotator at 4 °C for 30 min. After incubation, the MagBead-bound complex was washed with 19 μl Bead Binding Buffer, then with 19 μl Bead Wash Buffer and resuspended in 19 μl Bead Binding Buffer. The total amount of the MagBead-bound complex was loaded onto the machine. Seven SMRT cells were used for sequencing the poly(A)^+^ library and one for the random primer-based library.

### Read processing

Consensus reads were generated following the RS_ReadsOfInsert protocol of the SMRT Analysis (v2.3.0, patch 4), with the following settings: Minimum Full Passes=1, Minimum Predicted Accuracy=90, Minimum Length of Reads of Insert=1, Maximum Length of Reads of Insert=No Limit. The RS_Isoseq protocol was applied to classify (Minimum Sequence Length=100) and cluster read data (Estimated cDNA Size between 1 kbp~2 kbp, Minimum Quiver Accuracy To Classify An Isoform As HQ=0.99). These consensus reads were mapped using GMAP^11^, with the following settings: gmap -d Genome.fa --nofails -f samse File.fastq>Mapped_file.sam.

### Code availability

1. SMRT Analysis: http://www.pacb.com/products-and-services/analytical-software/smrt-analysis/ (version 2.3.0, patch 4)
2. GMAP: http://research-pub.gene.com/gmap/ (version 2015-12-31)
3. Samtools: http://www.htslib.org/download/ (version 1.6)
4. Custom routines were used to acquire the quality information presented in this data descriptor. The codes have been archived on Github (doi: 10.5281/zenodo.1034511).

## Data Records

All sequencing data have been uploaded to the European Nucleotide Archive under the project accession PRJEB22072 (Data Citation 1). These data contain: raw h5 files, consensus sequences in FastQ format and mapped reads (mapped to the hg19 and to the FJ616285.1 genome builds). All data can be used without restrictions.

## Technical Validation

The isolated RNA and reverse transcribed cDNA fractions were quantified by Qubit (Life Technologies) fluorometer. The conditions for primer annealing and binding of the polymerase were determined by PacBio’s Binding Calculator in RS Remote. The libraries were measured by an Agilent 2,100 bioanalyzer using the Agilent High Sensitivity DNA Kit. To confirm the strain of the virus, a BLAST^12^ search was conducted, where all reads were aligned against all the complete human betaherpesvirus 5 genomes in the NCBI database. The reads aligned to the FJ616285.1 genome showed the fewest mismatches (Table 5), therefore this genome build was used as a reference genome to analyse the data.

## Usage Notes

These datasets were primarily produced to discover HCMV transcripts and as such, it is suitable for validating transcript candidates or testing transcript discovery tools. The raw files can be used to improve base calling algorithms or to develop new tools processing raw PacBio files. FastQ and binary alignment (bam) files have also been uploaded for each SMRT cell to facilitate the usage of the data. The FastQ files can be mapped to any reference genome, while the bam files contain reads already aligned to the FJ616285.1 and hg19 genomes. These aligned files can be analysed using for example samtools^13^ and bedtools^14^ or visualized using e.g. IGV^15^ or Geneious^16^. The uploaded files are not trimmed, they contain terminal poly(A) sequences as well as the 5′ adapter (AGAGTACATGGG), which can be used to determine the orientations of the reads.

The isolate of the HCMV strain Towne sequenced in these experiments shows several mutations compared to the closest reference genome (FJ616285.1) available in public databases, the most important being that our isolate only contains varS of the two variants described to be present in the ATCC HCMV strain Towne virus stock (VR-977). This rearrangement is mentioned in the description of the FJ616285.1 genome build. The analysis of genetic variants detected in our isolate can be used to compare to genetic variants found in different HCMV strains or isolates.

## Additional information

**How to cite this article:** Balázs, Z. *et al.* Long-read sequencing of the human cytomegalovirus transcriptome with the Pacific Biosciences RSII platform. *Sci. Data* 4:170194 doi: 10.1038/sdata.2017.194 (2017).

**Publisher’s note:** Springer Nature remains neutral with regard to jurisdictional claims in published maps and institutional affiliations.

## Supplementary Material



## Figures and Tables

**Table 1:** Summary of the raw sequencing datasets.

**Run name**	**No. of reads**	**Coverage**	**Average aligning read length**	**Average mismatch frequency**	**Average indel frequency**
Run1	1,668	9.174	1293.294±7.539	0.07%±0.004%	0.395%±0.018%
Run2	1,531	8.680	1333.135±7.865	0.0785%±0.012%	0.425%±0.023%
Run3	2,274	12.673	1310.457±6.366	0.08%±0.007%	0.463%±0.018%
Run4	2,307	9.154	933.072±5.788	0.099%±0.014%	0.426%±0.019%
Run5	3,098	18.395	1396.243±5.137	0.083%±0.003%	0.618%±0.019%
Run6	3,814	22.439	1383.451±4.55	0.079%±0.004%	0.547%±0.018%
Run7	9,358	43.429	1091.287±3.022	0.14%±0.008%	0.734%±0.014%
Run8	9,036	40.450	1052.634±2.975	0.191%±0.013%	0.684%±0.014%
Total	33,086	164.394	1168.371±1.509	0.128%±0.004%	0.616%±0.007%
Run accessions are ENA Run accession IDs. All sequencing runs belong to the same sample: ERS1870077. Sample complex means the solution prepared from the library by the addition of the DNA/Polymerase binding solution. Three sample complexes were prepared from the poly(A)-selected library (polyA1–3), and one for the random library. Runs marked with an asterisk (*) contain samples from a separate experiment as well (see the Methods section for details).					

**Table 2:** Summary statistics of the ROIs.

**Accession**	**Definition**	**Mismatches**	**E-value**
FJ616285	Human herpesvirus 5 strain Towne	43,285	4.38E-05
GQ121041	Human herpesvirus 5 transgenic strain Towne	44,041	4.34E-05
KX544836	Human herpesvirus 5 isolate VR5201	86,370	4.38E-05
KF493877	Human herpesvirus 5 transgenic isolate Towne-BAC-der	88,787	6.68E-05
AC146851	Human Herpesvirus 5 Towne-BAC isolate	88,805	6.68E-05
KF493876	Human herpesvirus 5 transgenic isolate Towne-BAC_UL96_Mutant	89,227	6.68E-05
AY315197	Human herpesvirus 5 strain Towne	89,875	5.68E-05
KX101023	Human herpesvirus 5 strain Toledo/Towne Chimera 3	193,241	4.61E-05
KX101022	Human herpesvirus 5 strain Toledo/Towne Chimera 2	203,141	4.50E-05
AH013698	Human herpesvirus 5 strain Toledo	217,386	0.000171
Quality values have been determined using the RS_Isoseq protocol. In case of Run 4, the random library, full-length non-chimeric reads were called without requiring the presence of poly(A) tails.			

**Table 3:** Summary statistics of the sequencing reads which aligned to the human genome (hg19) from each SMRT cell.

**Run name**	**Run accession**	**Selection method**	**Date sequenced**	**Sample complex**	**Raw h5 files size (MB)**
Run1	ERR2106421	oligo(dT)	05-11-2016	PolyA1	4,357
Run2	ERR2106422	oligo(dT)	20-11-2016	PolyA1	3,911
Run3	ERR2106423	oligo(dT)	20-11-2016	PolyA1	4,878
Run4	ERR2106424	Ribodepletion	20-11-2016	Random	6,344
Run5	ERR2106425	oligo(dT)	25-11-2016	PolyA2	6,637
Run6	ERR2106426	oligo(dT)	25-11-2016	PolyA2	7,423
Run7	ERR2106427	oligo(dT)	04-12-2016	PolyA3	23,199
Run8	ERR2106428	oligo(dT)	04-12-2016	PolyA3	21,274
Average values are given together with s.e. values.					

**Table 4:** Summary statistics of the sequencing reads which aligned to the HCMV genome (FJ616285.1) from each SMRT cell.

**Run name**	**No. of ROI**	**No. full length non-chimeric reads**	**Mean full-length non-chimeric read length**	**Mean no. of passes**	**Mean read quality of insert**
Run1	6,898	6,277	1,346	25	98.99%
Run2	6,349	5,790	1,377	20	98.97%
Run3	9,706	8,800	1,347	21	99.00%
Run4	16,346	14,241	901	32	99.24%
Run5	13,099	11,647	1,504	27	99.09%
Run6	16,995	15,015	1,461	24	99.15%
Run7	43,682	30,058	1,213	18	98.20%
Run8	43,315	28,686	1,179	18	98.30%
Average values are given together with s.e. values.					

**Table 5:** BLAST results confirm the strain of the virus.

**Run name**	**No. of reads**	**Coverage**	**Average aligning read length**	**Average mismatch frequency**	**Average indel frequency**
Run1	5,670	0.002	1171.04±9.63	0.742%±0.06%	0.661%±0.035%
Run2	5,204	0.002	1201.064±10.349	0.785%±0.073%	0.761%±0.039%
Run3	8,072	0.003	1176.594±8.123	0.744%±0.043%	0.782%±0.034%
Run4	9,900	0.003	845.664±5.383	2.486%±0.059%	1.24%±0.035%
Run5	10,852	0.005	1329.305±7.035	0.697%±0.044%	0.862%±0.027%
Run6	14,240	0.006	1270.59±6.149	0.652%±0.02%	0.815%±0.026%
Run7	34,868	0.011	968.521±3.475	0.85%±0.027%	1.055%±0.018%
Run8	33,830	0.010	929.996±3.417	0.976%±0.033%	1.119%±0.02%
Total	122,636	0.040	1047.903±1.956	0.965%±0.015%	0.994%±0.009%
All sequencing reads were aligned against all the complete HCMV genomes in the NCBI database. The ten results with the fewest mismatches are shown.					
