# Corrigendum: Long-read sequencing of the human cytomegalovirus transcriptome with the pacific biosciences RSII platform

**DOI:** 10.1038/sdata.2018.32

**Published:** 2018-03-06

**Authors:** Zsolt Balázs, Dóra Tombácz, Attila Szűcs, Michael Snyder, Zsolt Boldogkői

*Scientific Data* 4:170194 doi: 10.1038/sdata.2017.194 (2017); Published: 19 December 2017; Updated: 6 March 2018

In the original version of the Data Descriptor the tables were presented in the incorrect order. This has now been corrected in the HTML and PDF versions.

